# Children with Intestinal Failure Maintain Their Renal Function on Long-Term Parenteral Nutrition

**DOI:** 10.3390/nu13103647

**Published:** 2021-10-18

**Authors:** Anat Guz Mark, Shelly Levi, Miriam Davidovits, Luba Marderfeld, Raanan Shamir

**Affiliations:** 1 Institute of Gastroenterology, Nutrition and Liver Diseases, Schneider Children’s Medical Center of Israel, Petach Tikva 4920235, Israel; lu.marderfeld@gmail.com (L.M.); raanan@shamirmd.com (R.S.); 2 Sackler Faculty of Medicine, Tel-Aviv University, Tel-Aviv 6997801, Israel; levi.shelly@gmail.com (S.L.); doctors@012.net.il (M.D.); 3 Institute of Pediatric Nephrology, Schneider Children’s Medical Center of Israel, Petach Tikva 4920235, Israel; 4 Nutrition and Dietetics Department, Schneider Children’s Medical Center of Israel, Petach Tikva 4920235, Israel

**Keywords:** total parenteral nutrition, complications, kidney, proteinuria, calciuria, follow up

## Abstract

Background: Long-term parenteral nutrition (PN) has been associated with renal complications, including hypercalciuria, nephrocalcinosis, proteinuria and reduced glomerular filtration rate (GFR). Pediatric data are scarce and mostly short-term. Our study aimed to evaluate renal complications in children with intestinal failure (IF) receiving long-term PN. Methods: A cross-sectional study was performed in a tertiary pediatric IF clinic of patients receiving home-PN treatment for more than 1 year. Data regarding medical background, anthropometrics, laboratory investigations and abdominal sonography were retrieved. Results: Complete data were available for 15 children (67% males), with a median age of 6 (range 1.5–15) years and a median (IQR) PN duration of 4 (1.5–6) years. Low-grade proteinuria was identified in 61% and microalbuminuria in 30% of the cohort. Hypercalciuria and hyperoxaluria were present in 50% and 46%, respectively. One patient had nephrocalcinosis. The estimated GFR was normal in all but one patient who had pre-existing kidney disease. Conclusions: Pediatric IF patients can present with preserved kidney function after years of PN treatment. Despite the high prevalence of hypercalciuria, nephrocalcinosis was not common. Base line and long-term monitoring of various aspects of renal function would be essential to characterize the effects of prolonged PN on kidney functions in pediatric patients.

## 1. Introduction

Intestinal failure (IF) is commonly defined as a critical reduction in gut function below the minimum necessary for the absorption of macronutrients and/or water and electrolytes, such that intravenous supplementation is required to maintain health and/or growth [1]. In clinical practice, chronic IF is defined by the need for PN for >60 days due to intestinal disease, dysfunction or resection [2]. In children from developed countries, the most common causes for IF are short bowel syndrome (SBS) following extensive bowel resection (due to necrotizing enterocolitis (NEC), intestinal atresia, gastroschisis); intestinal mucosal diseases (congenital diarrheal disorders); and disorders of intestinal dysmotility (pediatric intestinal pseudo-obstruction) [3].

Despite major advancements in the management and prognosis of pediatric IF over the last decades, long-term PN still poses a significant risk for complications, including infections, central venous catheter-related thrombosis, progressive liver disease, disturbances of fluid and electrolytes balance, renal disease, abnormalities of growth and metabolic bone disease [4,5].

Renal complications of long-term PN treatment may include hypercalciuria, nephrocalcinosis, proteinuria and reduced glomerular filtration rate (GFR), which were mostly described in adults [6,7]. Few pediatric studies have indicated renal impairment in children receiving long-term PN as well, including mainly decreased renal function in early studies [8] and high prevalence of proteinuria or hypercalciuria and nephrocalcinosis [9] in more recent studies [10,11].

Suggested mechanisms for renal impairment in pediatric patients with IF are multi-factorial and may include persistent intravascular volume depletion related to intestinal malabsorption, high prevalence of prematurity and complicated neonatal hospitalization, recurrent sepsis episodes, hemodynamic instability episodes, use of nephrotoxic agents, and high incidence of nephrocalcinosis [12,13].

Despite these many potential risk factors, in our current clinical practice treating pediatric patients with IF, significant renal impairment seems to be infrequent. This study was aimed to evaluate various potential renal complications in children receiving long-term PN treatment.

## 2. Materials and Methods

### 2.1. Study Design and Population

This study was a cross-sectional retrospective study performed between 5.2017 and 12.2018. The study population included patients treated at the intestinal failure and rehabilitation clinic in Schneider Children’s Medical Center of Israel, which is a tertiary referral center for pediatric IF. Criteria for inclusion were patients under the age of 18 years, with the diagnosis of IF and chronic home PN treatment, followed in the clinic for more than 1 year. The data were extracted from electronic medical records.

The study was approved by the institutional ethical review board (no. 0229-16-RMC).

### 2.2. Data and Study Variables

Background characteristics included patients’ age and medical history, the etiology of IF and the duration of home PN support. Data regarding PN included the volume, energy, electrolytes and macronutrient composition, and schedule of the PN treatment.

Patients’ anthropometric and blood pressure measurements were documented. Blood pressure was measured and interpreted according to the 2017 American Academy of Pediatrics guidelines [14].

Laboratory investigations included serum creatinine, serum electrolytes, renin and aldosterone levels at rest, vitamin D and parathyroid hormone (PTH) levels. Renal function, presented as estimated glomerular filtration rate (eGFR), was calculated from plasma creatinine level using the modified Schwartz formula [15].

Urinary biochemistry parameters, either from a urinary sample or from a 24-h urine collection (when available), included creatinine, electrolytes, protein, albumin, oxalate and citrate levels. The urinary excretion of protein, calcium, oxalate and citrate were calculated in relation to urinary creatinine. Normal values were adjusted for age [16,17,18,19]. Urine spot samples for calcium–creatinine ratios were collected at least twice in the study period, and the average result was calculated.

All laboratory investigations were obtained in the morning and after a night on PN infusion in patients not receiving PN daily.

Proteinuria was defined as protein to creatinine ratio of >0.2 mg/mg in children older than two years of age and >0.5 mg/mg in infants and toddlers from 6 to 24 months [17,20].

Urinary albumin levels between 30 and 300 mg albumin/gram creatinine were considered as moderately increased, levels >300 mg albumin/gram creatinine were considered severely increased [21].

A renal ultrasound examination was performed in all patients and evaluated by the same experienced radiologist.

### 2.3. Statistical Analysis

Statistical analysis was performed using IBM SPSS statistics 25 (Armonk NY: IBM Corp). Associations between categorical variables were determined by Pearson’s X^2^ test and Fisher’s exact test as appropriate. Scaled non-parametric variables were analyzed by Spearman’s and Mann–Whitney statistics tests. *p*-value ≤ 0.05 was considered significant.

## 3. Results

The study cohort included 15 patients who fulfilled the inclusion criteria, 67% males, with a median age of 6 (range 1.5–15) years. Patients’ characteristics are detailed in Table 1. The median (IQR) duration of home PN treatment was 4 (1.5–6) years. Etiologies of IF included seven patients with SBS, four patients with congenital enteropathies, three patients with motility disorders (including Hirschsprung’s disease and intestinal pseudo-obstruction) and one patient with autoimmune enteropathy. Three patients had a history of prematurity (ranged 28–35 weeks of gestation). Patient no. 6 was diagnosed with Megacystis Microcolon Intestinal Hypoperistalsis Syndrome (MMIHS) and had neurogenic bladder and dysplastic kidneys.

Most of the patients (73%) were treated with PN daily, two patients received PN infusion over 6 days a week, and two patients received PN over 3 days a week. The median (IQR) daily volume of the PN bags was 1200 (950–1550) mL. All patients used a cycling PN schedule at night, with a median (IQR) infusion duration of 11 (10–12) h. All consumed oral or enteral nutrition during daytime as tolerated.

PN provided median (IQR) energy per body weight of 50 (40.2–59.5) kcal/kg/day. The median (IQR) amino acids component was 1.8 (1.4–2.1) grams per kg of body weight, with a median (IQR) non-protein kilocalorie to nitrogen ratio (NPC:N) of 150 (139.5–160). The median (IQR) sodium content in PN was 5.3 (3.4–7.8) mEq per kg of body weight. PN regimen and composition of the study population are detailed in Supplementary Table S1.

Anthropometric and blood pressure measurements were performed in all patients. The median (IQR) weight for age z-score was −1.09 (−1.73–−0.59), and median (IQR) height for age z-score was −1.89 (−2.66–−0.69). Patients’ blood pressures were all within normal ranges adjusted for age, gender and height.

The results of the laboratory work-up are detailed in Table 2. In two patients, the urine tests were incomplete due to technical difficulties obtaining the specimens. In 5 of 13 patients (38%), urine was collected over 24 h (Patients no. 4, 6, 9, 11, 15.), and in eight patients (62%), urine tests were obtained from morning samples.

**Figure 1:**
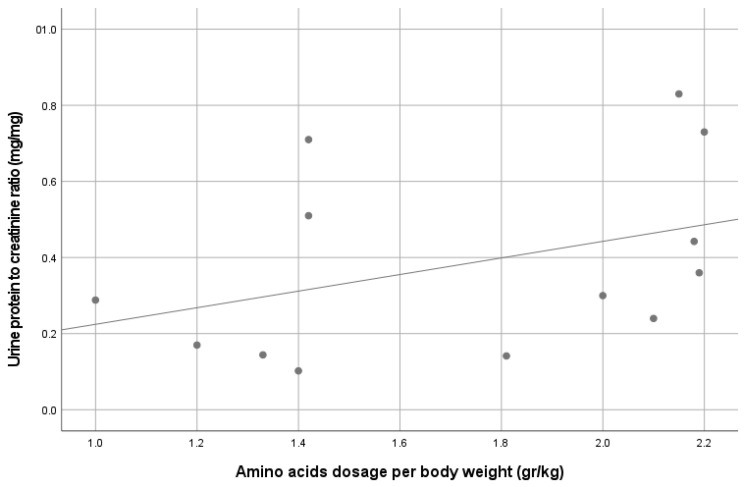
Correlation between urine protein-to-creatinine ratio and patients’ parenteral amino acid dosage. R = 0.56, *p* = 0.047.

**Figure 2:**
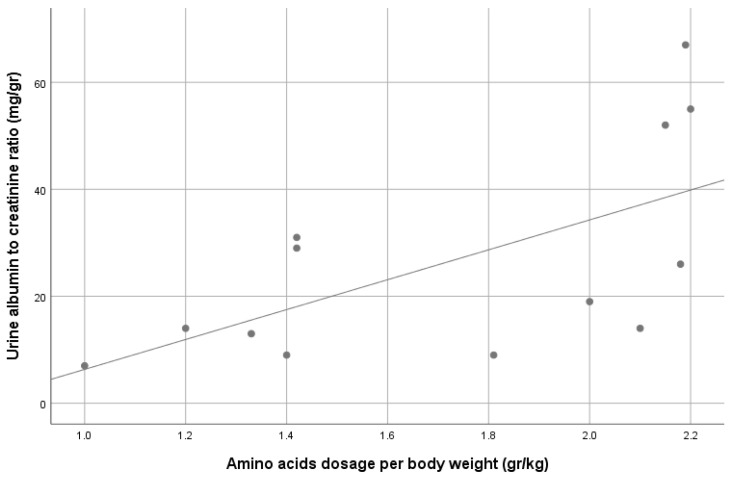
Correlation between urine albumin-to-creatinine ratio and patients’ parenteral amino acid dosage. R = 0.74, *p* = 0.004.

Proteinuria was observed in 8/13 (61%) patients, which was mostly low grade, and none had proteinuria within the nephrotic range. Four patients (30%) had microalbuminuria. A correlation was demonstrated between the parenteral amino acid dosage and both urine protein excretion (R = 0.56, *p* = 0.047) and urine albumin excretion (R = 0.74, *p* = 0.004), as demonstrated in Figure 1 and Figure 2, respectively. No other correlations were found between albuminuria or proteinuria and PN duration, PN volume or kcal, patients’ age and IF etiology (including SBS vs. other etiologies).

All but one patient had preserved renal function, as estimated by eGFR. The only patient with decreased eGFR of 82 L/min/1.73 m^2^, compatible with chronic kidney disease (CKD) grade 2 [22], was the patient with dysplastic kidneys and neurogenic bladder as a part of MMIHS (patient no. 6).

Urinary fractional excretion of sodium (FE_Na_) was below 1% in 13/14 (92%) of patients, suggesting a hypovolemic status. The urinary sodium to potassium ratio was highly variable.

Urine calcium to creatinine ratio was measured in 14 patients, 50% of them presenting hypercalciuria. Urine oxalate and urine citrate were assessed in 13 patients. Hyperoxaluria was observed in 46% of the patients and hypocitraturia in 30% of the patients.

Serum ionized calcium levels were all within the normal range during the study period. PTH level was elevated in 33% of patients; of them, two patients had PTH levels above 1.5 upper limits of normal (ULN). No patient had suppressed PTH values. Vitamin D deficiency (<50 nmol/liter) was observed in 33% of patients during the study period.

Renal ultrasound was normal in 12/15 (80%) patients. Abnormal findings included: hyperechogenic medulla compatible with medullary nephrocalcinosis and a small kidney stone in patient no. 14; cortical hyperechogenicity and decreased cortico-medullary differentiation in patient no. 15; and small echogenic dysplastic kidney with thin cortex and cortical cysts in patient no. 6 with MMIHS.

## 4. Discussion

This study provides a thorough investigation of potential renal complications among pediatric patients on long-term home PN treatment. The most remarkable finding in our study is the preserved renal function in this age group, despite significant intestinal morbidity, prolonged PN dependency and markers of hypovolemia.

A reduced GFR in the pediatric age group of patients receiving long-term PN was first reported by Moukarzel et al. [8]. In a small early study of 13 pediatric patients, published in 1991, all patients demonstrated a reduced GFR, which was inversely correlated with the duration of PN treatment. A decreased eGFR rate of 29% was also noted in a pediatric IF study conducted between 1990 and 2015, published by Ylinen et al. [23]. In Ylinen’s study, a decreased eGFR was associated with longer PN duration and a lower percentage of remaining small bowel length in SBS patients. In contrast to these early findings, the results from our study are in concordance with some more recent publications; a study by Messova et al. [24] found no significant deterioration of eGFR over a three-year period among 25 pediatric IF patients, and a study by Roberts et al. [11] also demonstrated preserved eGFR over a two-year period in pediatric IF. The discordance from studies demonstrating reduced renal function could be related to the era in which they were conducted, suggesting a more favorable outcome of home PN treatment nowadays. Another plausible explanation may lay in the fact that the patients in our study were generally less dependent on PN compared to the population of Ylinen’s study, the latter receiving a median of 7 weekly PN infusions and also included a portion of patients who underwent intestinal transplantation or were on a transplantation waiting list, possibly representing patients with more complicated disease course. In addition, the method used to evaluate renal function in our cohort, as well as in Messova’s and Roberts’ studies, was the eGFR calculated by Schwartz formula relaying on blood creatinine level measurement. As creatinine production correlates with muscle mass [25], using eGFR according to this method could cause overestimation of GFR in malnourished patients. This may explain the very high median (IQR) levels of eGFR levels of 172 (135–197) mL/min/1.73 m^2^ in our cohort.

A recent large study of adults patients on home PN treatment [26] showed that although reduced kidney function was frequent (in above 50% of the adult cohort), the probability of developing CKD during PN treatment was highest in those with decreased kidney function at the time of PN treatment initiation (which is more frequent in the adult population as well as with other comorbidities). This is in concordance with our study, in which the only patient with CKD had a pre-existing renal disease. Nevertheless, the possibility of a child on chronic PN treatment to develop renal impairment later in life should be of concern, considering the 2.8–3.5% annual decline of eGFR reported in adult studies [26].

Patients with IF could be at risk for hypovolemic state despite parenteral fluid support due to chronic diarrhea, high stoma fluid and electrolyte losses, and intestinal dysmotility. Lauverj at et al. [27] identified an association between decreased renal function in patients on long-term PN and signs of chronic dehydration, suggested by low urinary sodium to potassium ratio and high aldosterone and renin levels. Although the urinary sodium to potassium ratio was highly variable in our study, almost all patients had very low urinary FE_Na_ and high renin and aldosterone levels. These findings suggest a relative hypovolemic status, keeping in mind that laboratory investigations were obtained at morning time just after the period of PN infusion, which is probably the most well-hydrated time of the day in these patients. The low urinary FENa, combined with relatively high parenteral sodium supplementation given to the patients in this cohort, emphasize their significant fluid and electrolyte losses and support the need for high fluid and sodium supplementation in these conditions. Chronic hyperaldosteronism may cause kidney damage by itself [28] and should also be a matter of concern in the management of chronic IF. No correlation was found between the PN volume or sodium content per day and dehydration parameters.

Evidence of possible renal injury was observed by a high prevalence of proteinuria (61%) in our cohort, similar to the previous pediatric IF cohort published recently [10]. The degree of albuminuria was relatively low compared to the urine protein excretion, suggesting tubular proteinuria. Unfortunately, the assessment of tubular proteins (such as lysozyme or beta-2 microglobulin) was not available in this study. Although we could not find an association between proteinuria or albuminuria and the duration or amount of PN support, we found an association between those parameters and the amount of protein (amino acid dosage) supplied to these patients. Excessive consumption of protein was suggested to negatively impacted kidney function by a sustained increase in glomerular pressure and renal hyperfiltration [29]. The effects were documented to be related to the progression of renal disease in patients with CKD [30]. The negative effect of high protein meal on renal function might be further dependent on the volume status [31].

Long-term PN treatment, as well as malabsorption and IF, was associated with the risk for nephrocalcinosis and nephrolithiasis [9] by various mechanisms, including hyperoxaluria, hypercalciuria and hypocitraturia. Hyperoxaluria was noted in 46% of patients in our study. Intestinal malabsorption of lipids [32] and bile acids [33] facilitate the absorption of oxalate, and together with the PN solutions’ acidity and the presence of vitamin C may contribute to hyperoxaluria [34]. The nocturnal schedule of PN infusion could lead to a massive nocturnal loss of electrolytes, including calciuria [6]. In our study, half of the patients had hypercalciuria. These rates are in agreement with the results of a 60% incidence of calciuria in another pediatric cohort [10]. Furthermore, in concordance to previously pediatric series [35], hypocitraturia, which is a major risk factor for calcium stone formation [9,36], was diagnosed in 30% of our patients. The median urinary calcium to creatinine ratio in our calciuric patients was 1.87 times higher than the upper limit of normal for age. These values are lower than previously reported in IF patients with nephrocalcinosis [9]. As none of our patients in this cohort had suppressed PTH level, we assume that calciuria in a morning sample in those patients probably indicated the nocturnal parenteral calcium load rather than the diurnal calcium balance. As evidence of this, despite the high frequency of calciuria and hypocitraturia, nephrocalcinosis or renal stones were infrequent in our cohort. The rates of hypercalciuria, hypocitraturia and hyperoxaluria in our study were all slightly above the rates described recently in the pediatric cohort published by Roberts et al. [11], in which the patients were younger and with a lesser duration of chronic PN treatment. The latter study, however, reported a higher incidence of nephrocalcinosis than described in our study.

Although this study presents favorable outcomes in a pediatric IF population, it emphasizes the importance of tight monitoring and follow-up. Monitoring of renal complications is advisable as an integral part of long-term management of children on home PN treatment, including frequent blood and urine tests, periodic blood pressure assessment, renal sonography and pediatric nephrology consultation [13]. This surveillance is warranted especially in children with permanent IF, as renal injury in early life may predispose to kidney disease in adulthood [12], and the high rate of proteinuria and calciuria in our cohort suggest that the preserved renal function may not last into adulthood.

Several limitations to our study should be acknowledged. As this is a small cohort from a single center, the generalization of the results to a wider population is limited, although pediatric IF is still considered a rare disease. We also had some missing data due to the study’s retrospective nature, as well as to logistic challenges in obtaining urine samples from the young patients that were hard to overcome. Renal function was evaluated using serum creatinine measurement, as cystatin c was not available retrospectively. As pointed above, the interpretation of results could be influenced by our standard of care that includes close monitoring and PN regimen adjustment. Moreover, oral and enteral intake of fluids and nutrients, which is encouraged for all patients but could also influence results, was not evaluated in this study. Despite these important limitations, our study provides a detailed evaluation of various aspects of renal function in patients on home PN treatment for many years.

## 5. Conclusions

In conclusion, in the modern era and under close monitoring, pediatric patients on long-term PN treatment may have favorable renal outcomes. Among this age group, GFR is normal in the absence of pre-existing kidney disease, although mild proteinuria and albuminuria are common and require surveillance. Despite the high prevalence of hypercalciuria and hyperoxaluria, and markers suggestive of hypovolemia, nephrocalcinosis is rare. Thus, tight monitoring, maintenance of adequate hydration, regular surveillance of renal function and personalized adjustments of PN regimen are essential in the treatment of children with IF. Continued follow-up into adulthood and examining larger long-term cohorts are required to evaluate the impact of pediatric-onset IF on different aspects of kidney functions later in life.

## Figures and Tables

**Table 1:** Patients’ characteristics.

Patient No.	Intestinal Failure Etiology	Underlying Disease	Small Bowel Length (cm)	Age (Years)	Weight (kg)	Weight Z-Score	Height (cm)	Height Z-Score	Duration of HPN (Years)	Other Diagnoses
1	SBS	Midgut volvulus	60	4	16	0.243	96	−1	4	
2	Dysmotilty	Pediatric intestinal pseudo-obstruction	full	6	18	−1.31	106	−2.67	6	
3	Enteropathy	Tricho-hepatoenteric syndrome	full	2	10.5	−2.43	83	−1.98	2	
4	SBS	Bowel resection post Hirschsprung disease	80	6	19	−0.92	107	−1.89	6	
5	SBS	Midgut volvulus	20	4.5	17	−0.34	103	−0.99	4.5	Prematurity
6	Dysmotilty	MMIHS	full	11	35	−0.8	133	−2.17	11	Dysplastic kidneys
7	SBS	Intestinal atresia	10	1.5	11	−0.39	79	−0.11	1.5	Prematurity
8	Dysmotilty	Hirschsprung disease (total colonic)	full	8	19	−1.95	112	−2.81	8	Mowat-Wilson Syndrome
9	Enteropathy	Microvillus inclusion disease	full	15	45	−1.82	150	−2.65	15	
10	SBS	Midgut volvulus	20	8	22	−1.09	122	−0.68	4	
11	Enteropathy	Microvillus inclusion disease	full	12	22	−3.2	110	−5.14	1.5	
12	Enteropathy	Congenital diarrhea	full	9	25	−1.11	135	−0.16	1	
13	SBS	Gastroschisis	0	1.5	11	−0.79	76	−0.2	1.5	Prematurity
14	Immune dysregulation	Autoimmune Enteropathy	full	3	13	−0.25	74	−5.4	1.5	
15	SBS	Bowel resection post Hirschsprung disease	160	8	21	−1.64	125	−0.7	1	

PN = parenteral nutrition; HPN = home parenteral nutrition; SBS = short bowel syndrome; MMIHS = megacystis microcolon intestinal hypoperistalsis syndrome.

**Table 2:** Results of laboratory investigation.

Patient No.	eGFR (mL/min/1.73 m^2^)	FENa (%)	Renin (mcIU/mL)	Aldosterone (pmol/L)	uCa/Cr (mg/mg)	PTH (pg/mL)	25(OH) Vitamin D (nmol/L)	1,25 (2OH) Vitamin D (pmol/L)	UrOxalate/Cr (mmol/mol)	UrCitrate/Cr (mg/gr)	uPr/Cr (mg/mg)	uAlb/Cr (mg/gr)
1	142	0.25	**132**	359	**0.57**	**54.2**	**36.7**	99.2	76	635	**0.29**	7.00
2	175	0.18	**656.5**	387	0.12	**83**	73.3	200	52	362	**0.30**	19.00
3	127	2.40	**58.4**	**874**	**0.23**	17.9	69.4	187	73	556	**0.36**	**67.00**
4	158	0.07	**739.8**	449	**0.34**	20	64.4	56.5	43	489	0.10	9.00
5	203	0.18	**183.8**	**798**	**1.11**	25	56.5	184	**132**	**48**	**0.51**	29.00
6	**82**	0.24	**978.3**	**2770**	**0.39**	21	102	31.2	**102**	735	**0.71**	**31.00**
7	172	0.25	**181.5**	**1100**	0.31	33	123	88				
8	178		**73.6**	**661**		36	65.9	172				
9	129	0.4	**53.4**	**624**	0.16	**62**	**34.8**	162	**70**	121	0.14	13.00
10	112	0.55	**80.1**	266	**0.26**	32	68.3	114	**131**	**19**	0.14	9.00
11	216	0.30	**56.4**	361	0.09	**85**	**40.2**	153	70	**80**	**0.73**	**55.00**
12	207	0.99	14.6	168	0.17	18	64.7	151	**64**	985	0.17	14.00
13	224	0.35	**307.6**	**2660**	0.04	30	63.3	173	22.5	1435	0.44	26.00
14	170	0.28	38.5	**822**	**0.78**	18	**23.2**	76.4	97.2	820	**0.83**	**52.00**
15	191	0.83	**117.2**	**568**	0.08	**78**	**49.2**	170	**61**	**21**	**0.24**	14.00
Median (IQR)	171.7 (135.3–196.9)	0.29 (0.24–0.52)	98.6 (56.9–183.2)	624 (374–848)	0.25 (0.13–0.38)	32 (20.5–58.1	64.4 (44.7–68.8)	153 (93.6–172.5)	70 (61–97.2)	489 (80–735)	0.3 (0.17–0.51)	19 (13–31)

Abnormal values for age are mark in **bold**. eGFR = estimated glomerular filtration rate; FENa = fractional excretion of sodium; uCa/Cr = urinary calcium to creatinine ration; PTH = parathyroid hormone; UrOxalate/Cr = urinary oxalate to creatinine ratio; UrCitrate/Cr = urinary citrate to creatinine ratio; uPr/Cr = urinary proteine to creatinine ratio; uAlb/Cr = urinary albumin to creatinine ratio; IQR = inter-quartile range.

## Data Availability

Full data of this study are available from the corresponding author on request.

## Supplementary Materials

The following are available online at https://www.mdpi.com/article/10.3390/nu13103647/s1, Table S1: Parenteral nutrition (PN) regimen.
