# Supplementary material for: Children with Intestinal Failure Maintain Their Renal Function on Long-Term Parenteral Nutrition
Source: Nutrients. 2021 Oct 18;13(10):3647. doi: 10.3390/nu13103647 (PMC8539167; doi:10.3390/nu13103647)
Supplement: Supplementary file 1 [file nutrients-13-03647-s001.zip › nutrients-1395422-supplementary.pdf]

Supplementary Table S1. Parenteral nutrition (PN) regimen.

Patient no.	PN bag volume (ml)	Days on PN per week	duration of PN infusion (hours)	Weekly PN volume (ml)	PN volume/Kg/ day (ml)	PN volume/Kg/ week (ml)	Daily PN composition:		
							Energy/Kg (Kcl)	Amino-acids/Kg (Kcl)	Sodium/Kg (mEq)
1	800	3	10	2400	50	150.0	28	1.0	2.2
2	2000	7	12	14000	111	777.8	62.2	2.0	12.2
3	900	7	10	6300	86	600.0	56.5	2.2	5.7
4	1000	3	12	3000	53	157.9	41.2	1.4	5.3
5	900	6	9	5400	53	317.6	39.1	1.4	2.4
6	2700	7	14	18900	77	540.0	44.2	1.4	6.4
7	900	7	11	6300	82	572.7	59	2.0	4.1
8	1200	7	11	8400	63	442.1	50	1.8	2.4
9	2500	7	9	17500	55	388.9	35	1.3	2.8
10	1200	7	10	8400	55	381.8	48.6	1.8	4.5
11	1500	7	12	10500	68	477.3	60	2.2	5.5
12	1200	6	9	7200	48	288.0	33	1.2	4.0
13	1200	7	12	8400	109	763.6	65	2.2	9.1
14	1300	7	16	9100	100	700.0	67	2.2	10.0
15	1600	7	12	11200	76	533.3	52	2.1	9.5
